# Safety of Immunomodulatory Systemic Therapies Used in the Management of Immune-Related Cutaneous Adverse Events

**DOI:** 10.3390/ph16111610

**Published:** 2023-11-15

**Authors:** Stephanie L. Gu, Sandy Nath, Alina Markova

**Affiliations:** 1 Dermatology Service, Department of Medicine, Memorial Sloan Kettering Cancer Center, New York, NY 10065, USA; 2 Urgent Care Service, Memorial Sloan Kettering Cancer, New York, NY 10065, USA; 3 Department of Dermatology, Weill Cornell Medical College, New York, NY 10065, USA

**Keywords:** immune-related cutaneous adverse events, safety, biologics

## Abstract

Immune-related cutaneous adverse events (ircAEs) commonly occur in patients on treatment with immune checkpoint inhibitors and can significantly reduce patient quality of life. These are often treated with immunomodulatory agents, including glucocorticoids, immunosuppressants, and biologics. While often effective at managing symptoms, these therapies can cause several adverse events which may limit their use. In addition, immunomodulatory agents should be used with particular caution in patients receiving immunotherapy, as the efficacy of the oncologic regimen may potentially be undermined. In this review, we summarize the safety of systemic therapies that are used in the management of ircAEs, with a particular focus on the resultant risk of secondary tumor progression in patients with active cancer.

## 1. Introduction

Immune-related cutaneous adverse events (ircAEs) occur frequently in nearly half of patients on treatment with immune checkpoint inhibitors (ICIs), including PD(L)-1 and CTLA4 inhibitors [1]. Previous studies have shown that the development of immune-related adverse events (irAEs) can predict improved outcomes, underscoring the importance of properly managing these complications [2]. These are often managed effectively with immunomodulatory agents, but many of these therapeutics have been associated with a wide array of adverse events (AEs). In recent years, newer biologic therapies targeting cytokines implicated in the inflammatory process have emerged, boasting increasingly amenable safety profiles. However, these medications all have the potential to influence the immune response, which is especially important to consider among patients with active cancer. Previous studies have highlighted the integral role the immune system plays in both the body’s inherent anti-cancer mechanisms and in the action of ICI therapy. In this review, we discuss the safety of systemic ircAE immunomodulating therapies, including immunosuppressives and biologic agents, focusing particularly on the potential for these medications to affect the efficacy of ICIs.

## 2. Results and Discussion

### 2.1. Immunosuppressives

#### 2.1.1. Glucocorticoids

Glucocorticoids, including prednisone, prednisolone, methylprednisolone, and dexamethasone, are a class of drugs with structural similarity to endogenous cortisol. These medications have anti-inflammatory, immunosuppressive, and vasoconstrictive properties, which contribute to their widespread utility as a treatment for ircAEs of various phenotypes, including maculopapular rash, lichenoid eruptions, bullous pemphigoid, and psoriasiform rash [1]. However, these medications are recommended by the National Comprehensive Cancer Network (NCCN) for Common Terminology Criteria for Adverse Events (CTCAE) grade ≥ 2 ircAEs only, as long-term use can lead to a variety of complications [3].

Complications in numerous organ systems have been reported among patients receiving long-term glucocorticoid treatment and are typically more prominent with higher medication doses, especially at levels that are supra-physiologic. The odds ratio of new-onset diabetes mellitus in those receiving glucocorticoids has been reported at 1.5–2.5 [4]. Hyperglycemia has, in turn, been associated with an increased risk of death in multiple cancers, including liver, pancreas, ovary, colorectum, lung, bladder, and breast [5]. Osteoporosis, myopathy, and osteonecrosis occur frequently and can significantly reduce quality of life [6]. Cushingoid body habitus and cutaneous changes, including striae, hirsutism, and skin thinning, may develop in both adults and children, with children also at risk for growth retardation [7]. Secondary adrenal insufficiency can occur as a result of long-standing suppression of the hypothalamic-pituitary-adrenal (HPA) axis and poses a life-threatening risk to abrupt discontinuation of exogenous glucocorticoid treatment [8]. The risk of infection, hypertension, cataracts, and gastritis/gastric ulcer formation is also increased [9].

Patients administered steroids are uniquely at risk of undermining the efficacy of their antineoplastics, including immune checkpoint inhibitors (ICIs). Multiple studies have documented an association of steroid use with decreased progression-free survival, including a meta-analysis and in a dose-dependent manner [10,11,12,13]. An overarching limitation of current studies is their retrospective nature and associated confounding factors, such as discontinuation of immunotherapy or indication for steroid use. When Ricciuti and Petrelli applied subgroup analysis, the indication for steroid administration (palliative and/or treatment of brain metastasis vs treatment for immune-related adverse events [irAEs]) was informative [14,15]. Overall survival (OS) remained significantly decreased for those administered steroids for palliation and/or brain metastasis but not in those prescribed strictly for the treatment of irAEs. There remains the possibility that CPI benefits may be attenuated in this subgroup of irAEs as there is evidence that irAEs confer an improved prognosis [16]. Prospective studies are warranted. As a result of the numerous short- and long-term risks (as well as theoretical risks on decreased efficacy of immunotherapy), the American Society of Clinical Oncology (ASCO) 2021 guidelines recommend; “the lowest possible dose of steroids should be used for the shortest possible duration” [17]. Steroid-sparing therapeutic options remain a desirable and reasonable treatment for ircAEs.

#### 2.1.2. Cyclosporine

Cyclosporine is a calcineurin inhibitor that is FDA-approved for the prevention of solid organ transplant rejection and certain autoimmune diseases, including psoriasis and rheumatoid arthritis. This medication has also been used for the treatment of various irAEs, including lichenoid eruptions and toxic epidermal necrolysis [18,19,20]. While this use is off-label, the American Society of Clinical Oncology (ASCO) and NCCN recommend the use of cyclosporine for grade 4 Steven-Johnson Syndrome (SJS), toxic epidermal necrolysis (TEN), and drug-induced hypersensitivity syndrome (DIHS) that failed to respond to corticosteroids [3,21]. The NCCN also recommends cyclosporine for severe lichenoid and psoriasiform ircAEs [3].

However, cyclosporine use is associated with numerous AEs and is generally recommended for short-term use only. Hypertension, renal dysfunction, neurologic AEs, infection, hypertrichosis, gingival hyperplasia, fatigue, myalgia, cough, and dyspnea have all been reported in association with cyclosporine [22]. The risk of these AEs appears to be dose-dependent. In addition, patients on cyclosporine may be more predisposed to the development of malignancy. In preclinical models, cyclosporine exposure led to increased cell proliferation and alterations in cells, leading to increased invasiveness in certain cancer models, including adenocarcinoma, non-small cell lung cancer, and squamous cell carcinoma (SCC), but suppression of malignant progression in certain forms of breast cancer and oral SCC [23,24,25,26,27]. Real-world risk of malignancy in human models also appears to be elevated with cyclosporine use: in a 5-year study, investigators found that patients on cyclosporine for treatment of psoriasis were at increased risk of cutaneous malignancy, particularly squamous cell carcinoma [28]. However, the degree of cyclosporine exposure may affect this risk, as one study found that short-term use of cyclosporine did not increase rates of malignancy [29]. In addition, lymphoproliferative disorders, including EBV-positive lymphoma, have been associated with cyclosporine use, but these are primarily limited to small case series or case reports [30]. To date, there is no data in the literature on the influence of cyclosporine on ICI efficacy.

#### 2.1.3. Azathioprine

Azathioprine is a thiopurine that is FDA-approved for the treatment of rheumatoid arthritis and as prophylaxis for kidney transplant rejection. When used to treat irAEs, azathioprine is most often used for autoimmune hepatitis, although it can also be used for the treatment of ICI-induced arthritis or ircAEs [31]. The NCCN recommends azathioprine for severe lichenoid ircAEs [3].

AEs to azathioprine treatment occur in between 15–28% of patients and most often include nausea, fever, fatigue, arthralgias/myalgias, rash, hepatotoxicity, infection, and bone marrow suppression [32]. As an immunosuppressive, azathioprine may also increase the risk of malignancy. Although the FDA warns users of the risk of lymphoma and hepatosplenic T cell lymphoma (HSTCL) in patients with inflammatory bowel disease, the data on long-term cancer risk with azathioprine treatment is currently mixed [33,34,35]. However, there does appear to be an increased risk of cutaneous malignancy in particular, especially squamous cell carcinoma and keratoacanthoma [36]. To date, there is no literature on the influence of azathioprine on ICI efficacy. However, preclinical models have shown that exposure to 6-thioguanine, another thiopurine, increased tumor response to immune checkpoint blockade [37]. It is unclear if this effect is observed in patients, and a clinical trial (TEMPLE) investigating this relationship is ongoing (Clinicaltrials.gov identifier NCT05276284).

#### 2.1.4. Methotrexate

Methotrexate (MTX) is a dihydrofolate reductase inhibitor that is FDA-approved for the treatment of some hematologic malignancies, certain forms of arthritis, and psoriasis. It has also been used successfully off-label for a variety of irAEs, including bullous pemphigoid and psoriasis [38,39,40]. However, this is typically reserved for severe or recalcitrant cases [41]. The NCCN recommends MTX for severe lichenoid and psoriasiform ircAEs [3].

The most common AEs include transaminitis, nausea/vomiting, stomatitis, thrombocytopenia, rash, diarrhea, alopecia, photosensitivity, pancytopenia, and dizziness [42]. Long-term studies show that AEs occur in 61–95% of patients, although many of these are not treatment-limiting [22]. Given the risk of liver damage, patients are instructed to avoid alcohol consumption while on methotrexate. However, the potential for irreversible liver damage and fibrosis with methotrexate use is controversial [43]. In addition, the FDA warns about the increased risk for malignancy, particularly cutaneous and lymphoproliferative neoplasms [42]. The data on this is mixed and may vary based on the indication for which methotrexate is being used—some studies found no increased risk of malignancy among patients receiving methotrexate for psoriasis specifically, while others found increased risk of cutaneous malignancies with methotrexate use across all indications [44,45,46,47,48]. However, for patients on treatment with ICIs, some data suggests that methotrexate may actually increase anti-tumor response; in vitro experiments have demonstrated that methotrexate induces increased maturation of dendritic cells and greater stimulation of T cells [39,49]. In addition, treatment with methotrexate was associated with a longer time to cancer progression when compared to patients on treatment with anti-TNFa agents [39,49]. Further investigation into the synergistic effects of MTX and ICIs is needed, and a clinical trial evaluating the use of methotrexate in conjunction with ICIs for solid tumors is currently ongoing.

#### 2.1.5. Mycophenolate Mofetil

Mycophenolate mofetil (MMF) is an inosine monophosphate dehydrogenase inhibitor that is FDA-approved for the prophylaxis of organ rejection. Due to its immunosuppressive properties, it has also been used successfully off-label for the treatment of numerous autoimmune conditions, including pemphigoid diseases, psoriasis, and various other inflammatory dermatoses. MMF has also been used successfully for ircAEs, including bullous pemphigoid [40].

Common AEs with MMF include pain, abdominal pain, fever, headache, infection, anemia, leukopenia, hypertension, edema, diarrhea/nausea, infection, dyspnea, rash, and insomnia [50]. Long-term, real-world studies have similar safety profiles [51]. As with other immunosuppressants, MMF also increases the risk of infection. Progressive multifocal leukoencephalopathy (PML) was also reported in clinical trials, although studies have not found a significant association between PML and MMF [52]. In addition, the FDA warns users of increased risk of malignancy, as 0.4–1% of patients developed a lymphoproliferative disease in clinical trials. However, numerous studies have shown that MMF use does not increase and may, in fact, lower malignancy risk [53,54,55]. There is currently no published data on the influence of MMF on ICI efficacy.

#### 2.1.6. IVIG

Intravenous immunoglobulin is comprised of pooled human antibodies and is FDA-approved for humoral immunodeficiency, chronic immune thrombocytopenia purpura, and chronic inflammatory demyelinating polyneuropathy. In addition, due to its immunomodulatory properties and relatively favorable safety profile, it is also used frequently as a second- or third-line treatment in various dermatologic autoimmune diseases [56]. IVIG has also been used to treat numerous irAEs and is recommended by the American Society of Clinical Oncology for the treatment of steroid-refractory severe cutaneous adverse reactions secondary to ICI treatment (SCARs) [21]. In addition, the NCCN recommends IVIG for ICI-induced SJS and severe bullous eruptions [3].

Common AEs include headaches, fatigue, nausea, chills, fever, abdominal pain, anemia, epistaxis, and vomiting [57]. In addition, severe AEs, including acute renal failure, aseptic meningitis syndrome, hemolysis, elevations of blood pressure, and pulmonary adverse reactions, have all been reported [58].

The risk of cancer progression has not been associated with IVIG use. In contrast, IVIG has been shown to have antimetastatic effects and may lead to tumor regression. Mouse models injected with sarcoma or melanoma cells experienced significantly fewer lung metastases when concomitantly treated with IVIG [59]. In addition, IVIG has shown anti-angiogenic properties in both mice and humans [60]. Cases of patients experiencing tumor regression following treatment with IVIG have also been reported in the literature, but large studies examining its effects on tumor progression or response to antineoplastic treatment are lacking [61]. To date, there is no data in the literature on the influence of IVIG on ICI efficacy. However, preclinical models suggest that IVIG may increase T cell recognition of tumor cells through IgG binding to tumor surface antigens and subsequent dendritic cell activation [62]. It is also important to note that IVIG may bind to and interfere with ICI therapies and, therefore, should not be infused simultaneously.

#### 2.1.7. Hydroxychloroquine

Hydroxychloroquine is an immunomodulator and anti-parasitic medication that is used to treat uncomplicated malaria, rheumatoid arthritis, systemic lupus erythematosus, and chronic discoid lupus erythematosus. It has also been used successfully in various dermatologic conditions, including ICI-induced subacute cutaneous lupus [63,64,65].

Common AEs associated with hydroxychloroquine use include gastrointestinal symptoms, headaches, and rashes [66]. There does not appear to be an elevated risk of malignancy with hydroxychloroquine use [67,68,69]. In fact, numerous preclinical models have shown that hydroxychloroquine can induce tumor suppression and may help overcome chemotherapy resistance [70]. It is unclear if this is clinically significant, as studies have not found a reduction in absolute cancer occurrence with hydroxychloroquine use [69,71]. One study, however, did find that the use of this medication may reduce the risk of metastatic cancer and death [69]. Its effect on the anti-tumor efficacy of ICIs is mixed, with some in vitro and mouse studies supporting a synergistic relationship and others suggesting a reduction in anti-PD1 benefit with hydroxychloroquine administration [72,73,74].

#### 2.1.8. Dapsone

Dapsone is an anti-infective sulfone that is FDA-approved for the treatment of leprosy and dermatitis herpetiformis. It can also be used off-label for a number of inflammatory dermatoses and has been used successfully for the treatment of ircAEs, including Sweet syndrome and immunobullous eruptions [40,75,76,77,78]. Common adverse effects include anorexia, nausea, vomiting, headache, dizziness, tachycardia, anxiety, insomnia, and skin disorders. In addition, dapsone may cause methemoglobinemia or hemolysis in some patients and should be used with caution in patients with glucose-6-phosphate dehydrogenase deficiency [79]. Very rarely, patients can experience dapsone hypersensitivity syndrome (DHS), which is characterized by fever, rash, lymphadenopathy, hepatitis, and leukopenia. This occurs in an estimated 1.4% of patients taking dapsone and tends to manifest in the first 6 weeks of treatment. Symptoms typically improve once dapsone is discontinued, but an estimated 9.9% of cases are fatal [80]. There is no data on the effects of dapsone on ICI efficacy.

#### 2.1.9. Apremilast

Apremilast is a phosphodiesterase-4 inhibitor (PDE4) that is FDA-approved for the treatment of psoriasis and psoriatic arthritis. This medication has also been used successfully for the treatment of ICI-induced psoriasis and is recommended by the NCCN [3,38,81,82]. In clinical trials, patients receiving apremilast for psoriasis most often reported diarrhea (6%), nausea (7%), upper respiratory tract infections (6%), headache (4%), abdominal pain (2%) and vomiting (2%). Long-term studies have found similar rates of AEs [83]. The FDA warns of serious toxicities, including hypersensitivity reactions and depression. However, long-term studies have found that these serious AEs occur rarely [84]. In addition, apremilast appears to be safe for use in patients with prior or current malignancy [85]. In fact, preclinical models have found that inhibition of PDE4 reduced the progression of lung and prostate cancers and induced apoptosis of chronic lymphocytic leukemia and colorectal tumor cells [86]. However, the effect of blockade of PDE4 with apremilast specifically on cancer progression in the setting of ICI has not been investigated.

#### 2.1.10. Acitretin

Acitretin is a systemic vitamin A derivative (retinoid) that is FDA-approved for the treatment of psoriasis. It is also used frequently in psoriasis, psoriasiform, or lichenoid eruptions induced by systemic cancer treatments, including ICIs, and is recommended by the NCCN for moderate psoriasiform ircAEs and severe lichenoid ircAEs [3,82,87,88,89].

Common side effects of acitretin often resemble those of hypervitaminosis A syndrome and most often include flushing, psychiatric disturbances, headache, myopathy, thinning/exfoliation of the skin, capillary leak syndrome, skeletal abnormalities, xeropthalmia, and vision and hearing loss [90]. Patients may also experience hyperlipidemia or hepatotoxicity. However, there does not appear to be a significant risk of liver damage or uncontrolled hyperlipidemia with long-term use of low-dose acitretin [91,92]. Acitretin is also contraindicated in women who are pregnant or nursing due to the risk of fetal deformities. Acitretin has also been shown to reduce the frequency of BCC and SCC development in high-risk patients [93]. There is no data on the influence of acitretin on ICI efficacy.

### 2.2. Biologics

#### 2.2.1. Ustekinumab (Anti-IL12/23)

Ustekinumab, an anti-IL-12/23 monoclonal antibody, is FDA-approved for the treatment of plaque psoriasis and inflammatory bowel disease. It can also be used off-label for the treatment of ircAEs, especially psoriasiform eruptions. In clinical trials, patients receiving ustekinumab for psoriasis most often reported nasopharyngitis (8%), upper respiratory tract infections (5%), and headache (3%) [94]. Overall, the long-term safety of ustekinumab has been well established, with AEs occurring at a rate comparable to that seen with a placebo [95]. In addition, ustekinumab has one of the lowest risks of AEs when compared to other common treatments for psoriasis [96].

The FDA warns users about the increased risk of infection, although the risk appears to be lower than that of patients on other biologics or comparable treatments [97,98,99]. Providers are also advised to test for TB prior to initiation of ustekinumab, but appropriate prophylaxis for patients with latent disease significantly reduces the risk of reactivation [100]. A rare association between posterior reversible encephalopathy syndrome and ustekinumab use has also been reported, but the literature is limited to case reports [101].

Among patients with cancer, there is a concern for ustekinumab’s influence on anti-tumor efficacy, as both IL-12 and IL-23 play integral roles in cancer biology. In preclinical studies, IL-12 demonstrated anti-tumor effects, with data suggesting a potential for its synergistic use with T cell-based immunotherapies. IL-12 expressing modified CAR-T cells were effective against B cell lymphoma, ovarian cancer, and hepatocellular carcinoma. In addition, the combination of IL-12 with anti-PD-L1 boosted T cells increased tumor regression in mouse models [102,103]. IL-23 appears to have an opposing effect and tends to be elevated in tumors. In mouse models, blockade of IL-23 had synergistic effects with immune checkpoint blockade. However, the effects of IL-23 may vary based on cancer type, as other studies have found that IL-23 levels decreased with tumor progression in pancreatic cancer patients, and IL-23 was inhibitory toward premalignant oral lesions in mouse models. Blockade of both IL12 and 23 via inhibition of their shared p40 subunit led to tumor growth in one study and no tumor development in another, suggesting that the dominant effects of shared IL-12/23 blockade vary with the characteristics of the tumor [102,103]. Therefore, it is a plausible concern that blockade of IL12/23 with ustekinumab may increase the risk of tumor progression in certain cancer types. Long-term real-world studies have not found an increased risk of malignancy with the use of ustekinumab, but its influence on anti-tumor efficacy among patients on ICIs specifically has not yet been investigated [104].

#### 2.2.2. Dupilumab (Anti-IL4/13)

Dupilumab is a monoclonal antibody that blocks IL-4 and IL-13, which are commonly implicated in allergic diseases. This biologic is FDA-approved for the treatment of asthma, atopic dermatitis, and nasal polyps. Dupilumab has also been used successfully off-label for the treatment of ICI-induced eczematous, maculopapular, and lichenoid eruptions, bullous pemphigoid, and lichen planus pemphigoides [105,106,107,108] and is recommended by the NCCN for severe ICI associated pruritus [3]. In clinical trials, patients most often reported injection site reactions, conjunctivitis, and oral herpes [109]. Long-term safety evaluations have found similar types and rates of AEs [110].

Although increased cancer risk with dupilumab use has been raised as a concern, an analysis found no association between dupilumab use and short-term risk of developing a malignancy [111]. There have been some cases in the literature of chronic T cell lymphoma (CTCL) diagnosis following initiation of dupilumab, but it is possible that these patients were initially misdiagnosed with atopic dermatitis as clinically, this may present similarly to CTCL [112,113]. In preclinical studies, IL-4 and IL-13 have been shown to activate tumor-associated macrophages and myeloid-derived suppressor cells, which have pro-tumor effects. In addition, IL-4/13 are up-regulated in a variety of tumors, thus serving as a potential therapeutic target. When IL-4 and IL-13 inhibiting therapies were administered in human xenografts and mouse models, tumor growth decreased, and death of malignant cells was observed [114]. Therefore, it is possible that blockade with IL-4 and IL-13 with dupilumab may reduce tumor progression and growth, but further investigation is needed. Dupilumab has also been shown to reduce eosinophil levels. This has potentially relevant oncologic implications, as eosinophils play an integral role in immunotherapy and can enhance response to ICI treatment [115,116,117]. While 43.6% of patients treated with dupilumab for ircAE experienced subsequent progression of their tumor, there is no clear association as the majority of these patients had preceding ongoing disease progression, metastatic disease at baseline, or had received systemic steroids. In addition, the NCCN recommends the use of dupilumab for the management of ircAEs. Further prospective investigations on the influence of dupilumab on ICI efficacy are needed [115].

#### 2.2.3. Omalizumab (Anti-IgE)

Omalizumab is an anti-IgE humanized monoclonal antibody that is FDA-approved for asthma and chronic idiopathic urticaria refractory to antihistamine treatment. In clinical trials, patients receiving omalizumab most often experienced headaches, nasopharyngitis, sinusitis, upper respiratory tract infection, and arthralgias [118]. A meta-analysis of real-world use of omalizumab found that the average AE rate is 4.0%, and long-term safety evaluations have found that AEs occur at rates similar to that seen with placebo [119,120].

This therapy has also shown success in the treatment of moderate–severe cases of ICI-induced pruritus, eczema, urticaria, and bullous pemphigoid. In a cohort of 34 patients who were receiving omalizumab for ircAEs, no AEs were reported [121]. This study also found that concurrent oral use decreased from 50% to 9% of patients, which reduces the risk of steroid-associated complications [122]. In addition, the NCCN recommends omalizumab for the treatment of severe ICI-induced pruritus [3]. Concerns of increased malignancy risk with omalizumab have been raised, especially given the role that IgE plays in cancer surveillance [123]. In fact, some investigators have found that low IgE levels may predict a higher risk for cancer development [124,125]. Therefore, blockade of IgE with omalizumab may theoretically increase the risk of primary malignancy development or progression of existing tumors. However, a pooled analysis of patients on omalizumab in clinical trials showed no increased risk of malignancy [126]. The effect of omalizumab on ICI efficacy in patients on active treatment has not been evaluated, but the Society for Immunotherapy of Cancer (SITC) and NCCN currently recommends the use of omalizumab for the treatment of ircAEs [3,21,127,128].

#### 2.2.4. Benralizumab (Anti-IL5)

Benralizumab targets the IL-5 receptor and is FDA-approved as an add-on treatment for the maintenance of severe asthma with an eosinophilic phenotype. It has also been used successfully for the treatment of drug rash with eosinophilia and systemic symptoms (DRESS), atopic dermatitis, and AEs associated with the breast cancer therapy alpelisib [129,130,131]. Although there is little published data on this indication, benralizumab may also be useful for the treatment of ircAEs, as eosinophils are frequently elevated and may be implicated in the pathologic process [105].

In clinical trials, the most common adverse reactions reported by patients were headache (8%), pyrexia (3%), pharyngitis (5%), and hypersensitivity reactions including urticaria and rash (3%). The risk of these AEs did not appear to increase with longer time or greater exposure to benralizumab [132].

As with all immunomodulatory drugs, the effect on cancer risk must also be considered, especially among patients on treatment with ICIs. Incidence for malignancy among patients exposed to benralizumab was low in both short and long-term follow-up studies, and real-world risk did not appear to be elevated [133,134]. In preclinical models, IL-5 promoted mobilization of lung cancer cells, thus suggesting a potential antineoplastic benefit of IL-5 blockade. However, treatment with benralizumab also reduces eosinophils, which play an integral role in tumor surveillance [116]. The significance of these findings for patients is unclear, and further investigation is needed. The impact of benralizumab on ICI efficacy, in particular, has also not been studied.

#### 2.2.5. Tocilizumab (Anti-IL6)

Tocilizumab is an anti-IL-6 antibody that is FDA-approved for the treatment of various forms of arthritis, systemic sclerosis-associated interstitial lung disease, and cytokine release syndrome. It has also been used successfully for a wide array of immune-related AEs (irAEs), including ircAEs such as morphea and DRESS [135,136]. Tocilizumab may also have utility in the treatment of ircAEs of other morphologies, as IL-6 is frequently elevated in these patients [105].

In clinical trials, patients most often reported upper respiratory tract infection (7%), nasopharyngitis (7%), headache (7%), hypertension (6%), dizziness (3%), and bronchitis (3%) [137]. Psoriasiform, maculopapular, and urticarial rashes have also been reported with tocilizumab use [138,139]. The long-term adverse effect profile for tocilizumab is similar, and safety is well established [140,141].

IL-6 signaling also plays an integral role in cancer growth and development and may promote resistance to both checkpoint inhibitors and cytotoxic chemotherapy [142,143]. One preclinical study found that mouse models treated with concurrent anti-IL-6 antibodies experienced enhanced efficacy of anti-PD-L1 therapies [144]. In addition, treatment with IL-6 blockade reduced the occurrence of ICI-induced toxicities, suggesting that irAEs can be managed with this therapy without undermining the anti-tumor response [145]. The current literature suggests that the use of tocilizumab does not increase the risk of malignancy and is a promising adjunct to existing antineoplastics [146,147].

#### 2.2.6. Anti-TNFa Agents

Anti-TNFa agents, including monoclonal antibodies infliximab, adalimumab, and golimumab and receptor fusion protein etanercept, are FDA-approved for a variety of inflammatory disorders, including ankylosing spondylitis, psoriasis, hidradenitis suppurativa, inflammatory bowel disease, and various forms of arthritis. These therapies can also be used off-label for a variety of indications and have shown success for the treatment of maculopapular, psoriasiform, and lichenoid ircAEs as well as hidradenitis suppurative and pyoderma gangrenosum, which can occur with ICI treatment [1,41,77,148,149]. The NCCN also recommends the use of etanercept and other biosimilar agents for the treatment of SJS [3].

While effective for a wide variety of indications, anti-TNFa agents are associated with numerous adverse effects. Most commonly, patients report headaches, injection site or infusion reactions, rash, upper respiratory tract infections, sinusitis, nausea, and diarrhea [150]. Severe side effects, including infection, demyelinating disease, and cancer development, have also been reported in association with anti-TNFa use. Hepatotoxicity, heart failure, cytopenias, hypersensitivity, and lupus-like syndromes may also occur.

The risk for cancer development, especially lymphomas and cutaneous neoplasms, may also increase with anti-TNFa treatment, but this concern is controversial [151]. One study found a small but statistically significant increase in lymphoma risk with anti-TNFa monotherapy use, but other investigators have found no increase in any malignancy risk [152,153,154,155,156]. Due to these concerns, patients with cancer have historically been discouraged from treatment with these agents. The paradigm may be shifting as more data emerges. However, the current literature remains mixed. One study found that patients with high-grade irAEs treated with TNFa inhibitors had decreased survival compared to those treated with corticosteroids [157]. Other studies found no decrease in survival, with preclinical models also demonstrating an improvement in anti-tumor ICI efficacy with concurrent use of anti-TNFa agents [158,159,160,161]. In general, experts believe that short courses of anti-TNFa inhibitors among patients on ICI for cancer treatment are safe and effectively reduce the occurrence of irAEs [162].

#### 2.2.7. Rituximab

Rituximab is an anti-CD20 agent that is FDA-approved for Hodgkin’s Lymphoma, chronic lymphocytic leukemia, rheumatoid arthritis, some forms of vasculitis, and pemphigus vulgaris. Off-label, rituximab has been used successfully for a wide variety of irAEs, particularly bullous pemphigoid [163].

In clinical trials, patients receiving rituximab most often reported infusion-related reactions, infection, depression, herpes simplex, alopecia, fatigue, abdominal pain, conjunctivitis, dizziness, and headache [164]. However, these were often manageable and did not require discontinuation of treatment. Among patients receiving rituximab for bullous pemphigoid, adverse events occurred in 24% and included infections, anemia, neutropenia, syndrome of inappropriate antidiuretic hormone secretion (SIADH), pruritus, and tachycardia [165]. Relative to azathioprine and mycophenolate mofetil, rituximab as a treatment for pemphigus was associated with a greater risk of COVID-19, parasitic, and CMV infections within the first 12 months of treatment and pneumonia, osteomyelitis, and viral diseases extending beyond the first year of therapy [166]. Lending to its clinical utility, rituximab demonstrates a steroid-sparing effect and appears to be associated with fewer adverse events than treatment with conventional prednisone dosed for pemphigoid diseases [162,167].

Real-world use of rituximab across various indications was not associated with an increased risk of cancer [168,169]. B cells appear to play an integral role in the anti-tumor actions of immunotherapies, but it is unclear if their depletion has any implications, as one study found that B cell depletion or absence did not impact anti-tumor efficacy of PD-1 inhibitors [170,171]. The clinical significance of these findings is also unclear as the effect of B cell depletion on ICI efficacy in human patients has not been thoroughly investigated. Currently, ASCO and NCCN recommend the use of rituximab for the treatment of moderate to severe ICI-induced bullous pemphigoid [3].

#### 2.2.8. Anti-IL17 Agents

Monoclonal antibodies secukinumab, ixekizumab, and brodalumab are FDA-approved for the treatment of plaque psoriasis, psoriatic arthritis, ankylosing spondylitis, and non-radiographic axial spondylarthritis. Off-label, these agents can also be used for ircAEs, as IL-17 is often elevated among these patients and may play a pathogenic role. Successful treatment of ICI-associated psoriasiform eruptions with secukinumab and ixekizumab has been reported in the literature [172,173,174].

In clinical trials, patients most often reported nasopharyngitis, arthralgia, diarrhea, upper respiratory tract infection, rhinitis, urticaria, rhinorrhea, pharyngitis, and injection site reactions. Long-term follow-up of patients receiving these agents for various indications showed a similar adverse event profile and was generally favorable [175]. While the FDA warns of serious adverse events, including infection, tuberculosis, and Crohn’s disease, long-term analyses found that the incidence of these events is very low [176,177,178].

The risk of primary malignancy development or progression of existing cancer is also a concern among patients receiving anti-IL17 agents. The relationship between IL-17 and tumor progression is controversial and may vary based on cancer type or tumor stage [179,180,181]. Some preclinical models have suggested that blockade of IL17 enhances tumor response to anti-PD1 immunotherapy in colorectal cancers [182]. In addition, high IL-17 levels have been associated with poor response to therapy among patients with these tumors [183]. The clinical significance of these findings is unclear, but anti-IL17 agent use does not appear to be correlated with a higher risk of malignancy [175,184,185]. The impact of IL17 blockade on ICI anti-tumor efficacy in patients is similarly unclear, as discussion in the literature is currently limited to case reports. Among them, one patient experienced tumor progression following secukinumab treatment, while others were treated successfully without cancer progression [173,186,187].

#### 2.2.9. Anti-IL23 Agents

Anti-IL23 agents risankizumab, guselkumab, and tildrakizumab are FDA-approved for the treatment of psoriasis. Off-label, these medications have been used successfully for ICI-induced psoriasis or psoriasiform eruptions and psoriatic arthritis [188,189,190]. In clinical trials, patients most often experienced upper respiratory tract infections, headache, fatigue, injection site reactions, arthralgia, diarrhea, and tinea infections. In a 2-year follow-up study, 28.6% of patients on risankizumab for psoriasis experienced an adverse event, most commonly pharyngitis (11.9%) and headache (7.7%). No patients experienced any serious AEs, and none required treatment interruption as a result of AEs [191]. Guselkumab demonstrated similarly promising safety results—in a 5-year-old follow-up study, patients experienced AEs at rates lower than or comparable to that seen in the placebo group [192]. While the FDA warns users about the increased risk of hypersensitivity reactions, infections, and tuberculosis with the use of anti-IL23 agents, long-term follow-up studies have found that very few to no patients experienced opportunistic infections or active tuberculosis infections [192,193].

As discussed previously, IL-23 may have tumor-promoting effects. In preclinical models, blockade of the IL23 receptor with antibodies enhanced response to immunotherapy through destabilization of regulatory T cells [194]. In fact, blockade of IL-23 without concomitant antineoplastics may already yield therapeutic benefit—the sole administration of a neutralizing monoclonal antibody against IL-23 suppressed lung metastases in a mouse model of melanoma [195]. The clinical significance of these findings is unclear, as the influence of anti-IL23 on the anti-tumor efficacy of ICI treatment in patients has not been thoroughly investigated. However, in long-term safety trials, the use of anti-IL23 agents was not associated with increased rates of malignancy compared to those in general or psoriasis populations (Table 1 and Table 2) [193,196,197]. 

### 2.3. Limitations

While all the included medications have been extensively studied, their published use in patients with active cancer is limited. Further investigation is needed to assess the existence of AEs unique to the cancer population. Moreover, data on the influence of these therapies on cancer progression and ICI efficacy is either lacking or limited to low-quality studies, and prospective investigation is needed. Many biologic therapies included were also only recently approved, and therefore, long-term safety data is limited.

## 3. Conclusions

Immune-related cutaneous adverse events are a frequent and bothersome complication of ICI therapy. Effective management strategies are needed to improve patient quality of life and reduce ICI treatment interruptions. Several systemic therapies have been used successfully for ircAEs, but the safety of these medications remains a concern. The use of these immunomodulatory treatments requires discretion among patients with active or recent cancer, as the risk of reducing anti-tumor effects must be considered. To date, these ircAE therapies have not been shown with certainty to increase the risk of cancer progression, relapse, or primary cancer development. However, data is very limited, and further prospective investigation is needed to elucidate the safety profile of these therapies in this unique patient population.

## 4. Materials and Methods

An extensive search of the literature was conducted up until 1 July 2023 via PubMed, ScienceDirect, and Google Scholar for articles and textbooks related to the efficacy and safety of the following medications: glucocorticoids, cyclosporine, azathioprine, methotrexate, mycophenolate mofetil, hydroxychloroquine, dapsone, IVIG, apremilast, acitretin, ustekinumab, dupilumab, omalizumab, benralizumab, tocilizumab, infliximab, adalimumab, golimumab, etanercept, rituximab, secukinumab, ixekizumab, brodalumab, risankizumab, guselkumab, and tildrakizumab. These medications were included at the discretion of an experienced oncodermatologist, given their immunomodulatory mechanism of action and their use for the treatment of ircAEs. Search keywords included the drug name alone, the drug name + “safety”, and the drug name + “immune checkpoint”. Food and Drug Administration (FDA) labels of all included medications were reviewed and cited. Irrelevant articles were excluded by review of the article title and/or abstract. Additional literature cited in retrieved articles was also reviewed where relevant. All articles retrieved detailing the use of these medications for immune-related cutaneous adverse events were included.

## Figures and Tables

**Table 1:** The safety profile of common ircAE treatments.

Class	Medication	ircAE Phenotype Indications per NCCN Guidelines	Common AEs
**Steroids**	Glucocorticoids	Maculopapular, pruritus, bullous dermatitis, lichenoid	Hypertension, hyperglycemia, osteoporosis, peptic ulcers, cataracts, glaucoma, infections
**Immunosuppressives**	Cyclosporine	Bullous dermatitis, lichenoid, psoriasiform	Hypertension, renal dysfunction, infection, hypertrichosis, gingival hyperplasia, fatigue, myalgia, cough, dyspnea
	Azathioprine	Lichenoid	Nausea, fever, fatigue, arthralgias/myalgias, rash, hepatotoxicity, infection
	Methotrexate	Lichenoid, psoriasiform	Transaminitis, nausea/vomiting, stomatitis, thrombocytopenia, rash, diarrhea, alopecia, photosensitivity, pancytopenia, dizziness
**Immunomodulators**	IVIG	Bullous dermatitis	Headaches, fatigue, nausea, chills, fever, abdominal pain, epistaxis, vomiting
	Apremilast	Psoriasiform	Diarrhea, nausea, upper respiratory tract infections, headache, abdominal pain, vomiting
	Acitretin	Lichenoid, psoriasiform	Flushing, headache, myopathy, thinning of the skin, capillary leak syndrome, skeletal abnormalities, xeropthalmia, vision/hearing loss
**Biologics**	Dupilumab	Pruritus	Injection site reactions, conjunctivitis, oral herpes
	Omalizumab	Pruritus	Headache, upper respiratory tract infection, arthralgias
	Anti-TNFa (infliximab, adalimumab, golimumab, etanercept)	Bullous dermatitis	Headache, injection site reactions, rash, upper respiratory tract infections, nausea, diarrhea
	Rituximab	Bullous dermatitis	Infusion reactions, infection, alopecia, fatigue, abdominal pain, conjunctivitis, dizziness, headache

**Table 2:** Risk of malignancy progression while on ICI therapy associated with ircAE treatments.

Class	Medication	Risk of Progression of Existing Malignancy While on ICI	Level of Evidence
**Glucocorticoids**	Glucocorticoids	Increased	IV [15,198]
**Immunosuppressives**	Cyclosporine	NA	NA
	Azathioprine	NA	NA
	Methotrexate	Decreased	V [37,47]
	Mycophenolate Mofetil	NA	NA
	Hydroxychloroquine	Mixed	V [70,71,72]
**Immunomodulators**	Dapsone	NA	NA
	IVIG	NA	NA
	Apremilast	NA	NA
	Acitretin	NA	NA
**Biologics**	Ustekinumab	Mixed	V [100,101]
	Dupilumab	Mixed	V [2,112,113,114,115]
	Omalizumab	Decreased	V [2]
	Benralizumab	Mixed	V [114]
	Tocilizumab	Decreased	V [142,143]
	Anti-TNFa (infliximab, adalimumab, golimumab, etanercept)	Mixed	IV [152,153,154,155,156]
	Rituximab	Mixed	V [168,169]
	Anti-IL17 (secukinumab, ixekizumab, brodalumab)	Mixed	IV [171,184,185]
	Anti-IL23 (risankizumab, guselkumab, tildrakizumab)	Decreased	V [192,193]

## References

[1] Geisler A.N., Phillips G.S., Barrios D.M., Wu J., Leung D.Y.M., Moy A.P., Kern J.A., Lacouture M.E. (2020). Immune checkpoint inhibitor-related dermatologic adverse events. J. Am. Acad. Dermatol..

[2] Robesti D., Nocera L., Belladelli F., Schultz J.G., Fallara G., Marandino L., Raggi D., Montorsi F., Msaouel P., Necchi A. (2023). The immune-related adverse events paradox in locally advanced or metastatic urothelial cancer after atezolizumab immunotherapy: Analysis of individual patient data from IMvigor210 and IMvigor211 trials. BJU Int..

[3] NCCN Clinical Practice Guidelines in Oncology (NCCN Guidelines®) Management of Immunotherapy-Related Toxicities. https://www.nccn.org/professionals/physician_gls/pdf/immunotherapy.pdf.

[4] Clore J.N., Thurby-Hay L. (2009). Glucocorticoid-induced hyperglycemia. Endocr. Pract..

[5] Rao Kondapally Seshasai S., Kaptoge S., Thompson A., Di Angelantonio E., Gao P., Sarwar N., Whincup P.H., Mukamal K.J., Gillum R.F., Holme I. (2011). Diabetes mellitus, fasting glucose, and risk of cause-specific death. N. Engl. J. Med..

[6] Dykman T.R., Gluck O.S., Murphy W.A., Hahn T.J., Hahn B.H. (1985). Evaluation of factors associated with glucocorticoid-induced osteopenia in patients with rheumatic diseases. Arthritis Rheum..

[7] Chaudhry H.S., Singh G. (2023). Cushing Syndrome. StatPearls.

[8] Husebye E.S., Pearce S.H., Krone N.P., Kämpe O. (2021). Adrenal insufficiency. Lancet.

[9] Oray M., Abu Samra K., Ebrahimiadib N., Meese H., Foster C.S. (2016). Long-term side effects of glucocorticoids. Expert Opin. Drug Saf..

[10] Scott S.C., Pennell N.A. (2018). Early Use of Systemic Corticosteroids in Patients with Advanced NSCLC Treated with Nivolumab. J. Thorac. Oncol..

[11] Arbour K.C., Mezquita L., Long N., Rizvi H., Auclin E., Ni A., Martínez-Bernal G., Ferrara R., Lai W.V., Hendriks L.E.L. (2018). Impact of Baseline Steroids on Efficacy of Programmed Cell Death-1 and Programmed Death-Ligand 1 Blockade in Patients With Non-Small-Cell Lung Cancer. J. Clin. Oncol..

[12] Dearden H., Au L., Wang D.Y., Zimmer L., Eroglu Z., Smith J.L., Cuvietto M., Khoo C., Atkinson V., Lo S. (2021). Hyperacute toxicity with combination ipilimumab and anti-PD1 immunotherapy. Eur. J. Cancer.

[13] van Not O.J., Verheijden R.J., van den Eertwegh A.J.M., Haanen J., Aarts M.J.B., van den Berkmortel F., Blank C.U., Boers-Sonderen M.J., de Groot J.B., Hospers G.A.P. (2022). Association of Immune-Related Adverse Event Management With Survival in Patients With Advanced Melanoma. JAMA Oncol..

[14] Faje A.T., Lawrence D., Flaherty K., Freedman C., Fadden R., Rubin K., Cohen J., Sullivan R.J. (2018). High-dose glucocorticoids for the treatment of ipilimumab-induced hypophysitis is associated with reduced survival in patients with melanoma. Cancer.

[15] Petrelli F., Signorelli D., Ghidini M., Ghidini A., Pizzutilo E.G., Ruggieri L., Cabiddu M., Borgonovo K., Dognini G., Brighenti M. (2020). Association of Steroids use with Survival in Patients Treated with Immune Checkpoint Inhibitors: A Systematic Review and Meta-Analysis. Cancers.

[16] Eggermont A.M.M., Kicinski M., Blank C.U., Mandala M., Long G.V., Atkinson V., Dalle S., Haydon A., Khattak A., Carlino M.S. (2020). Association Between Immune-Related Adverse Events and Recurrence-Free Survival Among Patients With Stage III Melanoma Randomized to Receive Pembrolizumab or Placebo: A Secondary Analysis of a Randomized Clinical Trial. JAMA Oncol..

[17] Schneider B.J., Naidoo J., Santomasso B.D., Lacchetti C., Adkins S., Anadkat M., Atkins M.B., Brassil K.J., Caterino J.M., Chau I. (2021). Management of Immune-Related Adverse Events in Patients Treated With Immune Checkpoint Inhibitor Therapy: ASCO Guideline Update. J. Clin. Oncol..

[18] Fixsen E., Patel J., Selim M.A., Kheterpal M. (2019). Resolution of Pembrolizumab-Associated Steroid-Refractory Lichenoid Dermatitis with Cyclosporine. Oncologist.

[19] Randhawa M., Archer C., Gaughran G., Miller A., Morey A., Dua D., Yip D. (2019). Combined immune therapy grade IV dermatitis in metastatic melanoma. Asia Pac. J. Clin. Oncol..

[20] Chow K.V.C., O'Leary C., Paxton-Hall F., Lambie D., O'Byrne K. (2022). Pembrolizumab-induced toxic epidermal necrolysis: Case report. Oxf. Med. Case Rep..

[21] Brahmer J.R., Lacchetti C., Schneider B.J., Atkins M.B., Brassil K.J., Caterino J.M., Chau I., Ernstoff M.S., Gardner J.M., Ginex P. (2018). Management of Immune-Related Adverse Events in Patients Treated With Immune Checkpoint Inhibitor Therapy: American Society of Clinical Oncology Clinical Practice Guideline. J. Clin. Oncol..

[22] Balak D.M.W., Gerdes S., Parodi A., Salgado-Boquete L. (2020). Long-term Safety of Oral Systemic Therapies for Psoriasis: A Comprehensive Review of the Literature. Dermatol. Ther..

[23] Qin X., Chen Z. (2019). Metabolic dependence of cyclosporine A on cell proliferation of human non-small cell lung cancer A549 cells and its implication in post-transplant malignancy. Oncol. Rep..

[24] Hojo M., Morimoto T., Maluccio M., Asano T., Morimoto K., Lagman M., Shimbo T., Suthanthiran M. (1999). Cyclosporine induces cancer progression by a cell-autonomous mechanism. Nature.

[25] Abikhair M., Mitsui H., Yanofsky V., Roudiani N., Ovits C., Bryan T., Oberyszyn T.M., Tober K.L., Gonzalez J., Krueger J.G. (2016). Cyclosporine A immunosuppression drives catastrophic squamous cell carcinoma through IL-22. JCI Insight.

[26] Gao L., Dong J., Zhang N., Le Z., Ren W., Li S., Li F., Song J., Wang Q., Dou Z. (2019). Cyclosporine A Suppresses the Malignant Progression of Oral Squamous Cell Carcinoma in vitro. Anticancer Agents Med. Chem..

[27] Jiang K., He B., Lai L., Chen Q., Liu Y., Guo Q., Wang Q. (2012). Cyclosporine A inhibits breast cancer cell growth by downregulating the expression of pyruvate kinase subtype M2. Int. J. Mol. Med..

[28] Paul C.F., Ho V.C., McGeown C., Christophers E., Schmidtmann B., Guillaume J.C., Lamarque V., Dubertret L. (2003). Risk of malignancies in psoriasis patients treated with cyclosporine: A 5 y cohort study. J. Investig. Dermatol..

[29] Väkevä L., Reitamo S., Pukkala E., Sarna S., Ranki A. (2008). Long-term follow-up of cancer risk in patients treated with short-term cyclosporine. Acta Derm.-Venereol..

[30] Geller S., Xu H., Lebwohl M., Nardone B., Lacouture M.E., Kheterpal M. (2018). Malignancy Risk and Recurrence with Psoriasis and its Treatments: A Concise Update. Am. J. Clin. Dermatol..

[31] Remash D., Prince D.S., McKenzie C., Strasser S.I., Kao S., Liu K. (2021). Immune checkpoint inhibitor-related hepatotoxicity: A review. World J. Gastroenterol..

[32] Mohammadi O., Kassim T.A. (2023). Azathioprine. StatPearls.

[33] Jiyad Z., Olsen C.M., Burke M.T., Isbel N.M., Green A.C. (2016). Azathioprine and Risk of Skin Cancer in Organ Transplant Recipients: Systematic Review and Meta-Analysis. Am. J. Transpl..

[34] Fraser A.G., Orchard T.R., Robinson E.M., Jewell D.P. (2002). Long-term risk of malignancy after treatment of inflammatory bowel disease with azathioprine. Aliment. Pharmacol. Ther..

[35] Pasternak B., Svanström H., Schmiegelow K., Jess T., Hviid A. (2013). Use of azathioprine and the risk of cancer in inflammatory bowel disease. Am. J. Epidemiol..

[36] Cho H.G., Kuo K.Y., Xiao K., Batra P., Li S., Tang J.Y., Sarin K.Y. (2018). Azathioprine and risk of multiple keratinocyte cancers. J. Am. Acad. Dermatol..

[37] Nazerai L., Willis S.C., Yankilevich P., Di Leo L., Bosisio F.M., Frias A., Bertolotto C., Nersting J., Thastrup M., Buus S. (2023). Thiopurine 6TG treatment increases tumor immunogenicity and response to immune checkpoint blockade. Oncoimmunology.

[38] Nikolaou V., Sibaud V., Fattore D., Sollena P., Ortiz-Brugués A., Giacchero D., Romano M.C., Riganti J., Lallas K., Peris K. (2021). Immune checkpoint-mediated psoriasis: A multicenter European study of 115 patients from the European Network for Cutaneous Adverse Event to Oncologic Drugs (ENCADO) group. J. Am. Acad. Dermatol..

[39] Shi C.R., Otto T.S., Thompson L.L., Chang M.S., Reynolds K.L., Chen S.T. (2021). Methotrexate in the treatment of immune checkpoint blocker-induced bullous pemphigoid. Eur. J. Cancer.

[40] Wang J., Hu X., Jiang W., Zhou W., Tang M., Wu C., Liu W., Zuo X. (2023). Analysis of the clinical characteristics of pembrolizumab-induced bullous pemphigoid. Front. Oncol..

[41] Apalla Z., Rapoport B., Sibaud V. (2021). Dermatologic immune-related adverse events: The toxicity spectrum and recommendations for management. Int. J. Womens Dermatol..

[42] HIGHLIGHTS OF PRESCRIBING INFORMATION These Highlights Do Not Include All the Information Needed to Use METHOTREXATE TABLETS Safely and Effectively. See Full Prescribing Information for METHOTREXATE TABLETS. https://www.accessdata.fda.gov/drugsatfda_docs/label/2020/040054s015,s016,s017.pdf.

[43] Salliot C., van der Heijde D. (2009). Long-term safety of methotrexate monotherapy in patients with rheumatoid arthritis: A systematic literature research. Ann. Rheum. Dis..

[44] Chou Y.J., Wu C.Y., Pan T.Y., Wu C.Y., Chang Y.T. (2022). Risk of malignancy in patients with psoriasis receiving systemic medications: A nested case-control study. Dermatol. Ther..

[45] Polesie S., Gillstedt M., Schmidt S.A.J., Egeberg A., Pottegård A., Kristensen K. (2023). Use of methotrexate and risk of skin cancer: A nationwide case-control study. Br. J. Cancer.

[46] Fiorentino D., Ho V., Lebwohl M.G., Leite L., Hopkins L., Galindo C., Goyal K., Langholff W., Fakharzadeh S., Srivastava B. (2017). Risk of malignancy with systemic psoriasis treatment in the Psoriasis Longitudinal Assessment Registry. J. Am. Acad. Dermatol..

[47] Yan M.K., Wang C., Wolfe R., Mar V.J., Wluka A.E. (2022). Association Between Low-Dose Methotrexate Exposure and Melanoma: A Systematic Review and Meta-analysis. JAMA Dermatol..

[48] Polesie S., Gillstedt M., Sönnergren H.H., Osmancevic A., Paoli J. (2017). Methotrexate treatment and risk for cutaneous malignant melanoma: A retrospective comparative registry-based cohort study. Br. J. Dermatol..

[49] Bass A.R., Abdel-Wahab N., Reid P.D., Sparks J.A., Calabrese C., Jannat-Khah D.P., Ghosh N., Rajesh D., Aude C.A., Gedmintas L. (2023). Comparative safety and effectiveness of TNF inhibitors, IL6 inhibitors and methotrexate for the treatment of immune checkpoint inhibitor-associated arthritis. Ann. Rheum. Dis..

[50] CellCept®(Mycophenolate Mofetil Capsules) (Mycophenolate Mofetil Tablets). https://www.accessdata.fda.gov/drugsatfda_docs/label/2009/050722s021,050723s019,050758s019,050759s024lbl.pdf.

[51] Takeuchi T., Hashimoto H., Matsumoto M. (2022). Long-term safety and effectiveness of mycophenolate mofetil in adults with lupus nephritis: A real-world study in Japan. Mod. Rheumatol..

[52] Neff R.T., Hurst F.P., Falta E.M., Bohen E.M., Lentine K.L., Dharnidharka V.R., Agodoa L.Y., Jindal R.M., Yuan C.M., Abbott K.C. (2008). Progressive multifocal leukoencephalopathy and use of mycophenolate mofetil after kidney transplantation. Transplantation.

[53] O'Neill J.O., Edwards L.B., Taylor D.O. (2006). Mycophenolate mofetil and risk of developing malignancy after orthotopic heart transplantation: Analysis of the transplant registry of the International Society for Heart and Lung Transplantation. J. Heart Lung Transplant..

[54] Lok S.D., Wong A.W., Khor Y.H., Ryerson C.J., Johannson K.A. (2022). Malignancy Risk Associated With Mycophenolate Mofetil or Azathioprine in Patients With Fibrotic Interstitial Lung Disease. Chest.

[55] Hirunsatitpron P., Hanprasertpong N., Noppakun K., Pruksakorn D., Teekachunhatean S., Koonrungsesomboon N. (2022). Mycophenolic acid and cancer risk in solid organ transplant recipients: Systematic review and meta-analysis. Br. J. Clin. Pharmacol..

[56] Hoffmann J.H.O., Enk A.H. (2019). High-Dose Intravenous Immunoglobulin in Skin Autoimmune Disease. Front. Immunol..

[57] HIGHLIGHTS OF PRESCRIBING INFORMATION These Highlights Do Not Include All the Information Needed to Use Privigen Safely and Effectively. See Full Prescribing Information for Privigen. https://www.fda.gov/files/vaccines%2C%20blood%20%26%20biologics/published/Package-Insert---Privigen.pdf.

[58] Guo Y., Tian X., Wang X., Xiao Z. (2018). Adverse Effects of Immunoglobulin Therapy. Front. Immunol..

[59] Shoenfeld Y., Fishman P. (1999). Gamma-globulin inhibits tumor spread in mice. Int. Immunol..

[60] Yasuma R., Cicatiello V., Mizutani T., Tudisco L., Kim Y., Tarallo V., Bogdanovich S., Hirano Y., Kerur N., Li S. (2016). Intravenous immune globulin suppresses angiogenesis in mice and humans. Signal Transduct. Target. Ther..

[61] Sapir T., Shoenfeld Y. (2005). Uncovering the hidden potential of intravenous immunoglobulin as an anticancer therapy. Clin. Rev. Allergy Immunol..

[62] Carmi Y., Spitzer M.H., Linde I.L., Burt B.M., Prestwood T.R., Perlman N., Davidson M.G., Kenkel J.A., Segal E., Pusapati G.V. (2015). Allogeneic IgG combined with dendritic cell stimuli induce antitumour T-cell immunity. Nature.

[63] Liu R.C., Sebaratnam D.F., Jackett L., Kao S., Lowe P.M. (2018). Subacute cutaneous lupus erythematosus induced by nivolumab. Australas. J. Dermatol..

[64] Zitouni N.B., Arnault J.P., Dadban A., Attencourt C., Lok C.C., Chaby G. (2019). Subacute cutaneous lupus erythematosus induced by nivolumab: Two case reports and a literature review. Melanoma Res..

[65] Marano A.L., Clarke J.M., Morse M.A., Shah A., Barrow W., Selim M.A., Hall R.P., Cardones A.R. (2019). Subacute cutaneous lupus erythematosus and dermatomyositis associated with anti-programmed cell death 1 therapy. Br. J. Dermatol..

[66] Gisondi P., Piaserico S., Bordin C., Bellinato F., Tozzi F., Alaibac M., Girolomoni G., Naldi L. (2021). The safety profile of hydroxychloroquine: Major cutaneous and extracutaneous adverse events. Clin. Exp. Rheumatol..

[67] Mao I.C., Lin C.Y., Wu C.L., Kor C.T., Chang C.C. (2018). Hydroxychloroquine and risk of development of cancers: A nationwide population-based cohort study. Ther. Clin. Risk Manag..

[68] Vyas A., Gomez-Casal R., Cruz-Rangel S., Villanueva H., Sikora A.G., Rajagopalan P., Basu D., Pacheco J., Hammond G.R.V., Kiselyov K. (2022). Lysosomal inhibition sensitizes TMEM16A-expressing cancer cells to chemotherapy. Proc. Natl. Acad. Sci. USA.

[69] Fardet L., Nazareth I., Petersen I. (2017). Effects of chronic exposure of hydroxychloroquine/chloroquine on the risk of cancer, metastasis, and death: A population-based cohort study on patients with connective tissue diseases. Clin. Epidemiol..

[70] Verbaanderd C., Maes H., Schaaf M.B., Sukhatme V.P., Pantziarka P., Sukhatme V., Agostinis P., Bouche G. (2017). Repurposing Drugs in Oncology (ReDO)-chloroquine and hydroxychloroquine as anti-cancer agents. Ecancermedicalscience.

[71] Fang Y.F., Chen Y.F., Chung T.T., See L.C., Yu K.H., Luo S.F., Kuo C.F., Lai J.H. (2017). Hydroxychloroquine and risk of cancer in patients with primary Sjögren syndrome: Propensity score matched landmark analysis. Oncotarget.

[72] Vyas A., Cruz-Rangel S., Khan N., Bisignani M., Arantes L., Schmitt N., Kiselyov K., Ferris R., Duvvuri U. (2022). Hydroxychloroquine synergizes with anti-PD-1 immune checkpoint blockade in squamous carcinoma of the head and neck. J. ImmunoTherapy Cancer.

[73] Krueger J., Santinon F., Kazanova A., Issa M.E., Larrivee B., Kremer R., Milhalcioiu C., Rudd C.E. (2021). Hydroxychloroquine (HCQ) decreases the benefit of anti-PD-1 immune checkpoint blockade in tumor immunotherapy. PLoS ONE.

[74] Wabitsch S., McVey J.C., Ma C., Ruf B., Kamenyeva O., McCallen J.D., Diggs L.P., Heinrich B., Greten T.F. (2021). Hydroxychloroquine can impair tumor response to anti-PD1 in subcutaneous mouse models. iScience.

[75] Iriarte C., Young J.H., Rabin M.S., LeBoeuf N.R. (2022). Osimertinib-Induced Cutaneous Vasculitis Responsive to Low-Dose Dapsone Without Interruption of Anticancer Therapy: A Case Report and Review of the Literature. JTO Clin. Res. Rep..

[76] Asdourian M.S., Shah N., Jacoby T.V., Reynolds K.L., Chen S.T. (2022). Association of Bullous Pemphigoid With Immune Checkpoint Inhibitor Therapy in Patients With Cancer: A Systematic Review. JAMA Dermatol..

[77] Nazzaro G., Buffon S., Giacalone S., Maronese C.A., Marzano A.V. (2022). Skin manifestations associated with checkpoint inhibitors. JEADV Clin. Pract..

[78] Chen C.H., Yu H.S., Yu S. (2022). Cutaneous Adverse Events Associated with Immune Checkpoint Inhibitors: A Review Article. Curr. Oncol..

[79] PRESCRIBING INFORMATION INCLUDING PATIENT MEDICATION INFORMATION Pr MAR-DAPSONE. https://pdf.hres.ca/dpd_pm/00047474.PDF.

[80] Zhao Q., Sun L., Sun Y., Naisbitt D., Liu H., Zhang F. (2023). Dapsone hypersensitivity syndrome. Chin. Med. J. (Engl.).

[81] Mayor Ibarguren A., Enrique E.A., Diana P.L., Ana C., Pedro H.P. (2021). Apremilast for immune checkpoint inhibitor-induced psoriasis: A case series. JAAD Case Rep..

[82] Halle B.R., Betof Warner A., Zaman F.Y., Haydon A., Bhave P., Dewan A.K., Ye F., Irlmeier R., Mehta P., Kurtansky N.R. (2021). Immune checkpoint inhibitors in patients with pre-existing psoriasis: Safety and efficacy. J. Immunother. Cancer.

[83] Persson R., Cordey M., Paris M., Jick S. (2022). Safety of Apremilast in Patients with Psoriasis and Psoriatic Arthritis: Findings from the UK Clinical Practice Research Datalink. Drug Saf..

[84] Kavanaugh A., Gladman D.D., Edwards C.J., Schett G., Guerette B., Delev N., Teng L., Paris M., Mease P.J. (2019). Long-term experience with apremilast in patients with psoriatic arthritis: 5-year results from a PALACE 1-3 pooled analysis. Arthritis Res. Ther..

[85] Bernardini N., Skroza N., Marchesiello A., Mambrin A., Proietti I., Tolino E., Maddalena P., Marraffa F., Rossi G., Volpe S. (2022). Psoriatic patients with a history of cancer: A real-life experience with Apremilast treatment for 104 weeks. Dermatol. Ther..

[86] Peng T., Gong J., Jin Y., Zhou Y., Tong R., Wei X., Bai L., Shi J. (2018). Inhibitors of phosphodiesterase as cancer therapeutics. Eur. J. Med. Chem..

[87] Killion L., Beatty P., Byrne N., Mahon J.M., Salim A., Connolly M., Tobin A.M. (2021). Nivolumab Induced Psoriasis Successfully Treated With Acitretin. J. Drugs Dermatol..

[88] Masterson W.M., Brown A.M., Al Ameri M.A., Patel A.B. (2022). A retrospective chart review of management strategies for lichenoid eruptions associated with immune-checkpoint inhibitor therapy from a single institution. Cancer Treat. Res. Commun..

[89] Said J.T., Elman S.A., Perez-Chada L.M., Mita C., Merola J.F., LeBoeuf N.R. (2022). Treatment of immune checkpoint inhibitor-mediated psoriasis: A systematic review. J. Am. Acad. Dermatol..

[90] Zito P.M., Mazzoni T. (2023). Acitretin. StatPearls.

[91] Chularojanamontri L., Silpa-Archa N., Wongpraparut C., Limphoka P. (2019). Long-term safety and drug survival of acitretin in psoriasis: A retrospective observational study. Int. J. Dermatol..

[92] Hugh J., Van Voorhees A.S., Nijhawan R.I., Bagel J., Lebwohl M., Blauvelt A., Hsu S., Weinberg J.M. (2014). From the Medical Board of the National Psoriasis Foundation: The risk of cardiovascular disease in individuals with psoriasis and the potential impact of current therapies. J. Am. Acad. Dermatol..

[93] Allnutt K.J., Vogrin S., Li J., Goh M.S., Brennand S., Davenport R., Chong A.H. (2022). A long-term cohort study of acitretin for prevention of keratinocyte carcinoma in solid organ transplant recipients. Australas J. Dermatol..

[94] HIGHLIGHTS OF PRESCRIBING INFORMATION These Highlights Do Not Include All the Information Needed to Use STELARA® Safely and Effectively. See Full Prescribing Information for STELARA®. https://www.accessdata.fda.gov/drugsatfda_docs/label/2018/125261s147lbl.pdf.

[95] Sandborn W.J., Rebuck R., Wang Y., Zou B., Adedokun O.J., Gasink C., Sands B.E., Hanauer S.B., Targan S., Ghosh S. (2022). Five-Year Efficacy and Safety of Ustekinumab Treatment in Crohn's Disease: The IM-UNITI Trial. Clin. Gastroenterol. Hepatol..

[96] Daudén E., Carretero G., Rivera R., Ferrándiz C., Llamas-Velasco M., de la Cueva P., Belinchón I., Gómez-García F.J., Herrera-Acosta E., Ruiz-Genao D.P. (2020). Long-term safety of nine systemic medications for psoriasis: A cohort study using the Spanish Registry of Adverse Events for Biological Therapy in Dermatological Diseases (BIOBADADERM) Registry. J. Am. Acad. Dermatol..

[97] Jin Y., Lee H., Lee M.P., Landon J.E., Merola J.F., Desai R.J., Kim S.C. (2022). Risk of Hospitalization for Serious Infection After Initiation of Ustekinumab or Other Biologics in Patients With Psoriasis or Psoriatic Arthritis. Arthritis Care Res..

[98] Cheng D., Kochar B.D., Cai T., Ananthakrishnan A.N. (2022). Risk of Infections With Ustekinumab and Tofacitinib Compared to Tumor Necrosis Factor α Antagonists in Inflammatory Bowel Diseases. Clin. Gastroenterol. Hepatol..

[99] Schneeweiss M.C., Savage T.J., Wyss R., Jin Y., Schoder K., Merola J.F., Sidbury R., Oduol T., Schneeweiss S., Glynn R.J. (2023). Risk of Infection in Children With Psoriasis Receiving Treatment With Ustekinumab, Etanercept, or Methotrexate Before and After Labeling Expansion. JAMA Dermatol..

[100] Tsai T.F., Ho V., Song M., Szapary P., Kato T., Wasfi Y., Li S., Shen Y.K., Leonardi C. (2012). The safety of ustekinumab treatment in patients with moderate-to-severe psoriasis and latent tuberculosis infection. Br. J. Dermatol..

[101] Jordan A., Kinnucan J. (2022). Ustekinumab-Associated Posterior Reversible Encephalopathy Syndrome in a Patient With Crohn's Disease. ACG Case Rep. J..

[102] Mirlekar B., Pylayeva-Gupta Y. (2021). IL-12 Family Cytokines in Cancer and Immunotherapy. Cancers.

[103] Yan J., Smyth M.J., Teng M.W.L. (2018). Interleukin (IL)-12 and IL-23 and Their Conflicting Roles in Cancer. Cold Spring Harb. Perspect. Biol..

[104] Hong S.J., Zenger C., Pecoriello J., Pang A., Vallely M., Hudesman D.P., Chang S., Axelrad J.E. (2022). Ustekinumab and Vedolizumab Are Not Associated With Subsequent Cancer in IBD Patients with Prior Malignancy. Inflamm. Bowel Dis..

[105] Phillips G.S., Wu J., Hellmann M.D., Postow M.A., Rizvi N.A., Freites-Martinez A., Chan D., Dusza S., Motzer R.J., Rosenberg J.E. (2019). Treatment Outcomes of Immune-Related Cutaneous Adverse Events. J. Clin. Oncol..

[106] Shipman W.D., Singh K., Cohen J.M., Leventhal J., Damsky W., Tomayko M.M. (2023). Immune checkpoint inhibitor-bullous pemphigoid is characterized by interleukin-4 and interleukin-13 expression and responds to dupilumab treatment. Br. J. Dermatol..

[107] Pop S.R., Strock D., Smith R.J. (2022). Dupilumab for the treatment of pembrolizumab-induced bullous pemphigoid: A case report. Dermatol. Ther..

[108] Said J.T., Iriarte C., Talia J., Leung B., Virgen C.A., Robertson M., Rabin M.S., Larocca C., LeBoeuf N.R. (2023). Pembrolizumab-Associated Expansion of Radiation-Induced Morphea Responsive to Dupilumab: A Case Report. Clin. Exp. Dermatol..

[109] HIGHLIGHTS OF PRESCRIBING INFORMATION These Highlights Do Not Include All the Information Needed to Use DUPIXENT Safely and Effectively. See Full Prescribing Information for DUPIXENT. https://www.accessdata.fda.gov/drugsatfda_docs/label/2022/761055s040lbl.pdf.

[110] Blauvelt A., Guttman-Yassky E., Paller A.S., Simpson E.L., Cork M.J., Weisman J., Browning J., Soong W., Sun X., Chen Z. (2022). Long-Term Efficacy and Safety of Dupilumab in Adolescents with Moderate-to-Severe Atopic Dermatitis: Results Through Week 52 from a Phase III Open-Label Extension Trial (LIBERTY AD PED-OLE). Am. J. Clin. Dermatol..

[111] Owji S., Ungar B., Dubin D.P., Poplausky D., Young J.N., Ghalili S., Han J., Srinivasan D., Packer S., Pavel A.B. (2023). No association between dupilumab use and short-term cancer development in atopic dermatitis patients. J. Allergy Clin. Immunol. Pract..

[112] Elston D.M. (2020). Dupilumab and cutaneous T-cell lymphoma. J. Am. Acad. Dermatol..

[113] Russomanno K., Carver DeKlotz C.M. (2021). Acceleration of cutaneous T-cell lymphoma following dupilumab administration. JAAD Case Rep..

[114] Suzuki A., Leland P., Joshi B.H., Puri R.K. (2015). Targeting of IL-4 and IL-13 receptors for cancer therapy. Cytokine.

[115] Kuo A.M., Gu S., Stoll J., Moy A.P., Dusza S.W., Gordon A., Haliasos E.C., Janjigian Y., Kraehenbuehl L., Quigley E.A. (2023). Management of immune-related cutaneous adverse events with dupilumab. J. Immunother. Cancer.

[116] Grisaru-Tal S., Rothenberg M.E., Munitz A. (2022). Eosinophil-lymphocyte interactions in the tumor microenvironment and cancer immunotherapy. Nat. Immunol..

[117] Chu X., Zhao J., Zhou J., Zhou F., Jiang T., Jiang S., Sun X., You X., Wu F., Ren S. (2020). Association of baseline peripheral-blood eosinophil count with immune checkpoint inhibitor-related pneumonitis and clinical outcomes in patients with non-small cell lung cancer receiving immune checkpoint inhibitors. Lung Cancer.

[118] HIGHLIGHTS OF PRESCRIBING INFORMATION These Highlights Do Not Include All the Information Needed to Use XOLAIR Safely and Effectively. See Full Prescribing Information for XOLAIR. https://www.accessdata.fda.gov/drugsatfda_docs/label/2016/103976s5225lbl.pdf.

[119] Tharp M.D., Bernstein J.A., Kavati A., Ortiz B., MacDonald K., Denhaerynck K., Abraham I., Lee C.S. (2019). Benefits and Harms of Omalizumab Treatment in Adolescent and Adult Patients With Chronic Idiopathic (Spontaneous) Urticaria: A Meta-analysis of "Real-world" Evidence. JAMA Dermatol..

[120] Gevaert P., Saenz R., Corren J., Han J.K., Mullol J., Lee S.E., Ow R.A., Zhao R., Howard M., Wong K. (2022). Long-term efficacy and safety of omalizumab for nasal polyposis in an open-label extension study. J. Allergy Clin. Immunol..

[121] Barrios D.M., Phillips G.S., Geisler A.N., Trelles S.R., Markova A., Noor S.J., Quigley E.A., Haliasos H.C., Moy A.P., Schram A.M. (2021). IgE blockade with omalizumab reduces pruritus related to immune checkpoint inhibitors and anti-HER2 therapies. Ann. Oncol..

[122] Gutzmer R., Sibaud V., Hassel J.C. (2021). IgE blockade in the management of eosinophil-associated recalcitrant pruritus due to medical tumor therapy. Ann. Oncol..

[123] Jensen-Jarolim E., Achatz G., Turner M.C., Karagiannis S., Legrand F., Capron M., Penichet M.L., Rodríguez J.A., Siccardi A.G., Vangelista L. (2008). AllergoOncology: The role of IgE-mediated allergy in cancer. Allergy.

[124] Ferastraoaru D., Jordakieva G., Jensen-Jarolim E. (2021). The other side of the coin: IgE deficiency, a susceptibility factor for malignancy occurrence. World Allergy Organ. J..

[125] Ferastraoaru D., Bax H.J., Bergmann C., Capron M., Castells M., Dombrowicz D., Fiebiger E., Gould H.J., Hartmann K., Jappe U. (2020). AllergoOncology: Ultra-low IgE, a potential novel biomarker in cancer-a Position Paper of the European Academy of Allergy and Clinical Immunology (EAACI). Clin. Transl. Allergy.

[126] Busse W., Buhl R., Fernandez Vidaurre C., Blogg M., Zhu J., Eisner M.D., Canvin J. (2012). Omalizumab and the risk of malignancy: Results from a pooled analysis. J. Allergy Clin. Immunol..

[127] Ali Z., Egeberg A., Thyssen J.P., Sørensen J.A., Vestergaard C., Thomsen S.F. (2022). No association between omalizumab use and risk of cancer: A nationwide registry-based cohort study. Br. J. Dermatol..

[128] Bagnasco D., Canevari R.F., Del Giacco S., Ferrucci S., Pigatto P., Castelnuovo P., Marseglia G.L., Yalcin A.D., Pelaia G., Canonica G.W. (2022). Omalizumab and cancer risk: Current evidence in allergic asthma, chronic urticaria, and chronic rhinosinusitis with nasal polyps. World Allergy Organ. J..

[129] Lacouture M.E., Pan A., Dranitsaris G., Harris U., Chandarlapaty S., Dang C.T., Gajria D., Gordon A., Iyengar N.M., Robson M.E. (2022). Interim analysis of a single-center, single-arm, prospective phase 2 study to evaluate the efficacy and safety of benralizumab for alpelisib rash in metastatic PIK3CA-mutant, hormone receptor–positive breast cancer. J. Clin. Oncol..

[130] Maverakis E., Ji-Xu A., Brüggen M.C. (2022). Targeting interleukin-5 with benralizumab: A novel treatment for drug rash with eosinophilia and systemic symptoms. Allergy.

[131] Pham D.N. (2021). Spontaneous resolution of atopic dermatitis incidental to participation in benralizumab clinical trial for severe, uncontrolled asthma: A case report. J. Med. Case Rep..

[132] Korn S., Bourdin A., Chupp G., Cosio B.G., Arbetter D., Shah M., Gil E.G. (2021). Integrated Safety and Efficacy Among Patients Receiving Benralizumab for Up to 5 Years. J. Allergy Clin. Immunol. Pract..

[133] Jackson D.J., Korn S., Mathur S.K., Barker P., Meka V.G., Martin U.J., Zangrilli J.G. (2020). Safety of Eosinophil-Depleting Therapy for Severe, Eosinophilic Asthma: Focus on Benralizumab. Drug Saf..

[134] Mota D., Rama T.A., Moreira A. (2023). Real-world evidence on the risk of cancer with anti-IL-5 and anti-IL-4Ra biologicals. Allergy.

[135] Blaise M., Cardot-Leccia N., Seitz-Polski B., Picard-Gauci A., Bertold C., Passeron T., Montaudié H. (2023). Tocilizumab for Corticosteroid-Refractory Immune Checkpoint Inhibitor-Induced Generalized Morphea. JAMA Dermatol..

[136] Maximova N., Maestro A., Zanon D., Marcuzzi A. (2020). Rapid recovery of postnivolumab vemurafenib-induced Drug Rash with Eosinophilia and Systemic Symptoms (DRESS) syndrome after tocilizumab and infliximab administration. J. Immunother. Cancer.

[137] HIGHLIGHTS OF PRESCRIBING INFORMATION These Highlights Do Not Include All the Information Needed to Use ACTEMRA Safely and Effectively. See Full Prescribing Information for ACTEMRA. https://www.accessdata.fda.gov/drugsatfda_docs/label/2021/125276s131lbl.pdf.

[138] Palmou-Fontana N., Sánchez Gaviño J.A., McGonagle D., García-Martinez E., Iñiguez de Onzoño Martín L. (2014). Tocilizumab-induced psoriasiform rash in rheumatoid arthritis. Dermatology.

[139] Babkoor D., Alshuqayfi A., Alshegaifi N., Bamosa H., Alsaid M., Alkinani A., Algozi S., AlZaidi R., Alahmadi L., Hafiz W.A. (2022). Tocilizumab-Induced Dermatosis in a Patient With Rheumatoid Arthritis. Cureus.

[140] Khanna D., Lin C.J.F., Furst D.E., Wagner B., Zucchetto M., Raghu G., Martinez F.J., Goldin J., Siegel J., Denton C.P. (2022). Long-Term Safety and Efficacy of Tocilizumab in Early Systemic Sclerosis-Interstitial Lung Disease: Open-Label Extension of a Phase 3 Randomized Controlled Trial. Am. J. Respir. Crit. Care Med..

[141] Nishimoto N., Miyasaka N., Yamamoto K., Kawai S., Takeuchi T., Azuma J. (2009). Long-term safety and efficacy of tocilizumab, an anti-IL-6 receptor monoclonal antibody, in monotherapy, in patients with rheumatoid arthritis (the STREAM study): Evidence of safety and efficacy in a 5-year extension study. Ann. Rheum. Dis..

[142] Huseni M.A., Wang L., Klementowicz J.E., Yuen K., Breart B., Orr C., Liu L.F., Li Y., Gupta V., Li C. (2023). CD8(+) T cell-intrinsic IL-6 signaling promotes resistance to anti-PD-L1 immunotherapy. Cell Rep. Med..

[143] Chung A.W., Kozielski A.J., Qian W., Zhou J., Anselme A.C., Chan A.A., Pan P.Y., Lee D.J., Chang J.C. (2022). Tocilizumab overcomes chemotherapy resistance in mesenchymal stem-like breast cancer by negating autocrine IL-1A induction of IL-6. npj Breast Cancer.

[144] Mace T.A., Shakya R., Pitarresi J.R., Swanson B., McQuinn C.W., Loftus S., Nordquist E., Cruz-Monserrate Z., Yu L., Young G. (2018). IL-6 and PD-L1 antibody blockade combination therapy reduces tumour progression in murine models of pancreatic cancer. Gut.

[145] Hailemichael Y., Johnson D.H., Abdel-Wahab N., Foo W.C., Bentebibel S.E., Daher M., Haymaker C., Wani K., Saberian C., Ogata D. (2022). Interleukin-6 blockade abrogates immunotherapy toxicity and promotes tumor immunity. Cancer Cell.

[146] Kim S.C., Pawar A., Desai R.J., Solomon D.H., Gale S., Bao M., Sarsour K., Schneeweiss S. (2019). Risk of malignancy associated with use of tocilizumab versus other biologics in patients with rheumatoid arthritis: A multi-database cohort study. Semin Arthritis Rheum..

[147] Rubbert-Roth A., Sebba A., Brockwell L., Kelman A., Porter-Brown B., Pulley J., Napalkov P., van Vollenhoven R.F. (2016). Malignancy rates in patients with rheumatoid arthritis treated with tocilizumab. RMD Open.

[148] Maillard A., Pastor D., Merat R. (2021). Anti-PD-1-Induced Hidradenitis Suppurativa. Dermatopathology.

[149] Maronese C.A., Pimentel M.A., Li M.M., Genovese G., Ortega-Loayza A.G., Marzano A.V. (2022). Pyoderma Gangrenosum: An Updated Literature Review on Established and Emerging Pharmacological Treatments. Am. J. Clin. Dermatol..

[150] Gerriets V., Goyal A., Khaddour K. (2023). Tumor Necrosis Factor Inhibitors. StatPearls.

[151] Click B., Regueiro M. (2019). Managing Risks with Biologics. Curr. Gastroenterol. Rep..

[152] Lopez-Olivo M.A., Tayar J.H., Martinez-Lopez J.A., Pollono E.N., Cueto J.P., Gonzales-Crespo M.R., Fulton S., Suarez-Almazor M.E. (2012). Risk of Malignancies in Patients With Rheumatoid Arthritis Treated With Biologic Therapy: A Meta-analysis. JAMA.

[153] Lemaitre M., Kirchgesner J., Rudnichi A., Carrat F., Zureik M., Carbonnel F., Dray-Spira R. (2017). Association Between Use of Thiopurines or Tumor Necrosis Factor Antagonists Alone or in Combination and Risk of Lymphoma in Patients With Inflammatory Bowel Disease. JAMA.

[154] Williams C.J.M., Peyrin-Biroulet L., Ford A.C. (2014). Systematic review with meta-analysis: Malignancies with anti-tumour necrosis factor-α therapy in inflammatory bowel disease. Aliment. Pharmacol. Ther..

[155] Micic D., Komaki Y., Alavanja A., Rubin D.T., Sakuraba A. (2019). Risk of Cancer Recurrence Among Individuals Exposed to Antitumor Necrosis Factor Therapy: A Systematic Review and Meta-Analysis of Observational Studies. J. Clin. Gastroenterol..

[156] Mercer L.K., Galloway J.B., Lunt M., Davies R., Low A.L., Dixon W.G., Watson K.D., Symmons D.P., Hyrich K.L. (2017). Risk of lymphoma in patients exposed to antitumour necrosis factor therapy: Results from the British Society for Rheumatology Biologics Register for Rheumatoid Arthritis. Ann. Rheum. Dis..

[157] Verheijden R.J., May A.M., Blank C.U., Aarts M.J.B., van den Berkmortel F., van den Eertwegh A.J.M., de Groot J.W.B., Boers-Sonderen M.J., van der Hoeven J.J.M., Hospers G.A. (2020). Association of Anti-TNF with Decreased Survival in Steroid Refractory Ipilimumab and Anti-PD1-Treated Patients in the Dutch Melanoma Treatment Registry. Clin. Cancer Res..

[158] Bertrand F., Montfort A., Marcheteau E., Imbert C., Gilhodes J., Filleron T., Rochaix P., Andrieu-Abadie N., Levade T., Meyer N. (2017). TNFα blockade overcomes resistance to anti-PD-1 in experimental melanoma. Nat. Commun..

[159] Lesage C., Longvert C., Prey S., Maanaoui S., Dréno B., Machet L., Zehou O., Kramkimel N., Jeudy G., Skowron F. (2019). Incidence and Clinical Impact of Anti-TNFα Treatment of Severe Immune Checkpoint Inhibitor-induced Colitis in Advanced Melanoma: The Mecolit Survey. J. Immunother..

[160] Favara D.M., Spain L., Au L., Clark J., Daniels E., Diem S., Chauhan D., Turajlic S., Powell N., Larkin J.M. (2020). Five-year review of corticosteroid duration and complications in the management of immune checkpoint inhibitor-related diarrhoea and colitis in advanced melanoma. ESMO Open.

[161] Badran Y.R., Cohen J.V., Brastianos P.K., Parikh A.R., Hong T.S., Dougan M. (2019). Concurrent therapy with immune checkpoint inhibitors and TNFα blockade in patients with gastrointestinal immune-related adverse events. J. Immunother. Cancer.

[162] Chen A.Y., Wolchok J.D., Bass A.R. (2021). TNF in the era of immune checkpoint inhibitors: Friend or foe?. Nat. Rev. Rheumatol..

[163] Deftereos S.N., Georgonikou D. (2021). Effectiveness of rituximab in treating immune-checkpoint-inhibitor-induced immune-related adverse events: Results of a systematic review. Ann. Oncol..

[164] HIGHLIGHTS OF PRESCRIBING INFORMATION These Highlights Do Not Include All the Information Needed to Use RITUXAN Safely and Effectively. See Full Prescribing Information for RITUXAN. https://www.accessdata.fda.gov/drugsatfda_docs/label/2021/103705s5464lbl.pdf.

[165] Kremer N., Snast I., Cohen E.S., Hodak E., Mimouni D., Lapidoth M., Mazor S., Levi A. (2019). Rituximab and Omalizumab for the Treatment of Bullous Pemphigoid: A Systematic Review of the Literature. Am. J. Clin. Dermatol..

[166] Kridin K., Mruwat N., Amber K.T., Ludwig R.J. (2023). Risk of infections in patients with pemphigus treated with rituximab vs. azathioprine or mycophenolate mofetil: A large-scale global cohort study. Br. J. Dermatol..

[167] Frampton J.E. (2020). Rituximab: A Review in Pemphigus Vulgaris. Am. J. Clin. Dermatol..

[168] Alping P., Askling J., Burman J., Fink K., Fogdell-Hahn A., Gunnarsson M., Hillert J., Langer-Gould A., Lycke J., Nilsson P. (2020). Cancer Risk for Fingolimod, Natalizumab, and Rituximab in Multiple Sclerosis Patients. Ann. Neurol..

[169] van Daalen E.E., Rizzo R., Kronbichler A., Wolterbeek R., Bruijn J.A., Jayne D.R., Bajema I.M., Rahmattulla C. (2017). Effect of rituximab on malignancy risk in patients with ANCA-associated vasculitis. Ann. Rheum. Dis..

[170] Griss J., Bauer W., Wagner C., Simon M., Chen M., Grabmeier-Pfistershammer K., Maurer-Granofszky M., Roka F., Penz T., Bock C. (2019). B cells sustain inflammation and predict response to immune checkpoint blockade in human melanoma. Nat. Commun..

[171] Damsky W., Jilaveanu L., Turner N., Perry C., Zito C., Tomayko M., Leventhal J., Herold K., Meffre E., Bosenberg M. (2019). B cell depletion or absence does not impede anti-tumor activity of PD-1 inhibitors. J. Immunother. Cancer.

[172] Gleason L., Hunter E., Cohen A., Suriano J., Nikbakht N. (2023). Atezolizumab-induced psoriasiform drug eruption successfully treated with ixekizumab: A case report and literature review. Dermatol. Online J..

[173] Johnson D., Patel A.B., Uemura M.I., Trinh V.A., Jackson N., Zobniw C.M., Tetzlaff M.T., Hwu P., Curry J.L., Diab A. (2019). IL17A Blockade Successfully Treated Psoriasiform Dermatologic Toxicity from Immunotherapy. Cancer Immunol. Res..

[174] Kost Y., Mattis D., Muskat A., Amin B., McLellan B. (2022). Immune Checkpoint Inhibitor-Induced Psoriasiform, Spongiotic, and Lichenoid Dermatitis: A Novel Clinicopathological Pattern. Cureus.

[175] Gottlieb A.B., Deodhar A., McInnes I.B., Baraliakos X., Reich K., Schreiber S., Bao W., Marfo K., Richards H.B., Pricop L. (2022). Long-term Safety of Secukinumab Over Five Years in Patients with Moderate-to-severe Plaque Psoriasis, Psoriatic Arthritis and Ankylosing Spondylitis: Update on Integrated Pooled Clinical Trial and Post-marketing Surveillance Data. Acta Derm. Venereol..

[176] Deodhar A., Mease P.J., McInnes I.B., Baraliakos X., Reich K., Blauvelt A., Leonardi C., Porter B., Das Gupta A., Widmer A. (2019). Long-term safety of secukinumab in patients with moderate-to-severe plaque psoriasis, psoriatic arthritis, and ankylosing spondylitis: Integrated pooled clinical trial and post-marketing surveillance data. Arthritis Res. Ther..

[177] Schreiber S., Colombel J.F., Feagan B.G., Reich K., Deodhar A.A., McInnes I.B., Porter B., Das Gupta A., Pricop L., Fox T. (2019). Incidence rates of inflammatory bowel disease in patients with psoriasis, psoriatic arthritis and ankylosing spondylitis treated with secukinumab: A retrospective analysis of pooled data from 21 clinical trials. Ann. Rheum. Dis..

[178] Elewski B.E., Baddley J.W., Deodhar A.A., Magrey M., Rich P.A., Soriano E.R., Soung J., Bao W., Keininger D., Marfo K. (2021). Association of Secukinumab Treatment With Tuberculosis Reactivation in Patients With Psoriasis, Psoriatic Arthritis, or Ankylosing Spondylitis. JAMA Dermatol..

[179] Zou W., Restifo N.P. (2010). TH17 cells in tumour immunity and immunotherapy. Nat. Rev. Immunol..

[180] Kryczek I., Wei S., Szeliga W., Vatan L., Zou W. (2009). Endogenous IL-17 contributes to reduced tumor growth and metastasis. Blood.

[181] Zhao J., Chen X., Herjan T., Li X. (2020). The role of interleukin-17 in tumor development and progression. J. Exp. Med..

[182] Liu C., Liu R., Wang B., Lian J., Yao Y., Sun H., Zhang C., Fang L., Guan X., Shi J. (2021). Blocking IL-17A enhances tumor response to anti-PD-1 immunotherapy in microsatellite stable colorectal cancer. J. Immunother. Cancer.

[183] Pellegrini C., Esposito M., Rossi E., Gisondi P., Piaserico S., Dapavo P., Conti A., Gambardella A., Burlando M., Narcisi A. (2022). Secukinumab in Patients with Psoriasis and a Personal History of Malignancy: A Multicenter Real-Life Observational Study. Dermatol. Ther.

[184] Lebwohl M., Deodhar A., Griffiths C.E.M., Menter M.A., Poddubnyy D., Bao W., Jehl V., Marfo K., Primatesta P., Shete A. (2021). The risk of malignancy in patients with secukinumab-treated psoriasis, psoriatic arthritis and ankylosing spondylitis: Analysis of clinical trial and postmarketing surveillance data with up to five years of follow-up. Br. J. Dermatol..

[185] Deodhar A.A., Combe B., Accioly A.P., Bolce R., Zhu D., Gellett A.M., Sprabery A.T., Burmester G.R. (2022). Safety of ixekizumab in patients with psoriatic arthritis: Data from four clinical trials with over 2000 patient-years of exposure. Ann. Rheum. Dis..

[186] Ma V.T., Lao C.D., Fecher L.A., Schiopu E. (2022). Successful use of secukinumab in two melanoma patients with immune checkpoint inhibitor-induced inflammatory arthropathy. Immunotherapy.

[187] Esfahani K., Miller W.H. (2017). Reversal of Autoimmune Toxicity and Loss of Tumor Response by Interleukin-17 Blockade. N. Engl. J. Med..

[188] Takeda K., Yanagitani N. (2022). Guselkumab for treating immune checkpoint inhibitor-induced psoriatic arthritis. Ann. Rheum. Dis..

[189] Gargiulo L., Ibba L., Valenti M., Costanzo A., Narcisi A. (2023). Pembrolizumab-induced plaque psoriasis successfully treated with risankizumab in a patient with stage IV cutaneous melanoma. Melanoma Res..

[190] Glinos G.D., Fisher W.S., Morr C.S., Seminario-Vidal L. (2021). Nivolumab-induced psoriasis successfully treated with risankizumab-rzaa in a patient with stage III melanoma. JAAD Case Rep..

[191] Megna M., Ruggiero A., Battista T., Marano L., Cacciapuoti S., Potestio L. (2023). Long-Term Efficacy and Safety of Risankizumab for Moderate to Severe Psoriasis: A 2-Year Real-Life Retrospective Study. J. Clin. Med..

[192] Lebwohl M.G., Merola J.F., Rowland K., Miller M., Yang Y.W., Yu J., You Y., Chan D., Thaçi D., Langley R.G. (2023). Safety of Guselkumab Treatment for up to 5 Years in Patients With Moderate-to-Severe Psoriasis: Pooled Analyses Across Seven Clinical Trials With Greater Than 8600 Patient-Years of Exposure. Br. J. Dermatol..

[193] Gordon K.B., Lebwohl M., Papp K.A., Bachelez H., Wu J.J., Langley R.G., Blauvelt A., Kaplan B., Shah M., Zhao Y. (2022). Long-term safety of risankizumab from 17 clinical trials in patients with moderate-to-severe plaque psoriasis. Br. J. Dermatol..

[194] Wight A.E., Sido J.M., Degryse S., Ao L., Nakagawa H., Qiu Vivian Y., Shen X., Oseghali O., Kim H.J., Cantor H. (2022). Antibody-mediated blockade of the IL23 receptor destabilizes intratumoral regulatory T cells and enhances immunotherapy. Proc. Natl. Acad. Sci. USA.

[195] Teng M.W., von Scheidt B., Duret H., Towne J.E., Smyth M.J. (2011). Anti-IL-23 monoclonal antibody synergizes in combination with targeted therapies or IL-2 to suppress tumor growth and metastases. Cancer Res..

[196] Blauvelt A., Lebwohl M., Langley R.G., Rowland K., Yang Y.W., Chan D., Miller M., You Y., Yu J., Thaҫi D. (2023). Malignancy rates through 5 years of follow-up in patients with moderate-to-severe psoriasis treated with guselkumab: Pooled results from the VOYAGE 1 and VOYAGE 2 trials. J. Am. Acad. Dermatol..

[197] Thaci D., Piaserico S., Warren R.B., Gupta A.K., Cantrell W., Draelos Z., Foley P., Igarashi A., Langley R.G., Asahina A. (2021). Five-year efficacy and safety of tildrakizumab in patients with moderate-to-severe psoriasis who respond at week 28: Pooled analyses of two randomized phase III clinical trials (reSURFACE 1 and reSURFACE 2). Br. J. Dermatol..

[198] Ricciuti B., Dahlberg S.E., Adeni A., Sholl L.M., Nishino M., Awad M.M. (2019). Immune Checkpoint Inhibitor Outcomes for Patients With Non-Small-Cell Lung Cancer Receiving Baseline Corticosteroids for Palliative Versus Nonpalliative Indications. J. Clin. Oncol..

